# An Atypical Suicide Attempt: Self-Inflicted Intra-Cardiac Injury with Sewing Needle

**Published:** 2014

**Authors:** Nazila Shahmansouri, Mahmood Shirzad, Sam Zeraatiannejad Davani, Fetemeh Heidari

**Affiliations:** 1 Assistant Professor, Department of Psychiatry, Tehran Heart Center, Tehran University of Medical Sciences, Tehran, Iran.; 2 Assistant Professor, Department of Cardiovascular Surgery, Tehran Heart Center, Tehran University of Medical Sciences, Tehran, Iran.; 3 Cardiac Surgery Fellowship, Department of Cardiovascular Surgery, Tehran Heart Center, Tehran University of Medical Sciences, Tehran, Iran.; 4 Psychologist, Tehran Heart Center, Research Branch, Tehran, Iran.

**Keywords:** Atypical Suicide, Self-Inflicted Injury, Surgery

## Abstract

Atypical cases of suicide are less likely to be seen in general hospitals, nonetheless require further investigation into the precipitating factors as well as proper follow-up. This paper illustrates a 61-year-old woman with major depressive disorder who experienced auditory hallucinations during delirious periods of taking low-dose benzodiazepines, who referred to the hospital with a sewing needle stuck in her chest wall. The needle was successfully removed. Psychiatric problems are often underdiagnosed, therefore undertreated in general hospitals. Thus close monitoring of the patients during the hospitalization and after discharge is crucial.

## Introduction

The two most common suicide methods are self-poisoning and self-injury, respectively (1). Suicidal attempt is a risk factor for successful suicide (2). Studies have shown that 24-42% of those who attempted suicide had planned in less than 5 minutes (2). In some studies, young age and history of physical illness in females have been demonstrated as suicide risk factors (3). Many suicide victims suffer from physical illness and psychiatric disorders (2).

Psychiatric problems are often underdiagnosed, and therefore, undertreated in general hospitals (4, 5). Moreover, unconventional suicide methods can increase diagnostic and therapeutic complications. This paper reports a rare suicide method (penetrating sharp foreign bodies into the heart) of which only a few similar cases were found in the literature (6, 7). Most cases of self-harm are demonstrations of underlying psychiatric problems (8).

## Case Report

A. P., a 61-year-old woman, was taken to the emergency ward of the Tehran Heart Center, Iran, by her husband and son. On admission, she was suffering from dyspnea, and there was a sewing needle penetrating her chest. During the physical examination, the heart and lungs were normal. Emergency fluoroscopy, echocardiography and computed tomography (CT) scan were performed. The chest CT scan revealed that the needle had penetrated into the heart, and echocardiography demonstrated that the needle had ruptured the pericardium, but it had not entered the left ventricle. The patient underwent surgery 4 hours after admission, and the needle was removed successfully.

An interview with her family disclosed a history of major depressive disorder and pharmacological treatment. During the first 3 sessions of psychiatric counseling after the surgical operation, the patient denied a suicide attempt. She claimed that an accidental fall was the cause of her injury. On the 4^th^ session, however, she admitted that the problem was not accidental, stating that she had attempted unplanned impulsive suicide by swallowing needles twice before, once 10 years and once a year previous to this. In both cases, she had been hospitalized for some days and discharged without any complications.

The patient had been suffering from major depressive disorder since the sudden death of her son, 10 years ago. During the previous 5 years, she had received different pharmacological treatments, namely clonazepam, alprazolam, venlafaxine, and Depakine, which were not followed through. No history of psychotherapy or electroconvulsive therapy was reported.

The patient reported having experienced auditory hallucinations during different periods of taking low-dose benzodiazepine pills. According to her family, during these periods, the patient had experienced disorientation, disturbed consciousness, distraction, and auditory hallucinations. Through these accounts we were guided toward the delirious picture of those episodes in her past. She also self-medicated with benzodiazepine pills in those periods reported by the family, as she was not receiving adequate attention from them.

The family members described her as a demanding and irritable person who needs a great deal of attention, and is also prone to impulsive behavior when under stress or not receiving adequate attention.

After thorough examination, venlafaxine was prescribed as antidepressant. The patient should have remained hospitalized until the completion of the treatment, but the patient as well as her family members consented to leave the hospital after 5 days despite the advice of the medical team. Efforts to convince her to adhere to the course of treatment failed due to her refusal to do so.

## Discussion

Violent methods of suicide attempts are uncommon. In this case, the patient initially denied having attempted suicide, but finally disclosed her previous suicide attempts and depressive symptoms.

Our patient's bizarre attempt at taking her own life could be explained in the following two ways:

1. Her personality traits of being demanding, impulsive, and sensitive to self-perceived inadequate attention might have rendered her prone to these attempts. This is evidenced by her impulsive and self-prescribed consumption of benzodiazepine pills as a result of feelings ignored as reported by her family and significant other. Usually, individuals with higher impulsivity are at a greater risk of attempting suicide (9).
2. Swallowing needles or sticking them into the chest wall could be an instance of self-harm inasmuch as an impulsive person is liable to exhibit self-harming behavior (9). Nevertheless, the possibility of suicide attempts should not be discounted.

Unfortunately, our patient's refusal to permit follow-up prevented us from further looking into this unusual case. This study highlights the necessity of a multi-disciplinary approach to examining suicide cases. It is advisable to consider surgical cases which appear unusual to the surgeon as possible instances of self-harm or suicide attempts and to place them under close clinical observation. In general hospitals, the psychiatric background of the patient is likely to be overlooked, and as a result, suitable treatment is not provided (5). In most regions of the world, less than half of the suicide cases are estimated to receive the necessary medical attention (10). Alarmingly, this is inconsistent with the finding that these individuals tend to have further self-harming attempts in their lives (11). Patients with no previous suicidal ideation, depressive mood, or hopelessness, no history of substance or alcohol abuse, and strong social network and support are at lower risk of attempting suicide (12). Close monitoring of patients during hospitalization is crucial. The majority of successful suicides occur shortly after hospital discharge (13). Ignoring patients' psychiatric history and impulsive suicidal attempt after discharge lead to the underestimation of the suicide risk of patients admitted to general hospitals (13). 

## Notes

**Declaration of interest:** None.
